# Fractional Flow Reserve versus Angiography–Guided Management of Coronary Artery Disease: A Meta–Analysis of Contemporary Randomised Controlled Trials

**DOI:** 10.3390/jcm11237092

**Published:** 2022-11-30

**Authors:** Annette M. Maznyczka, Connor J. Matthews, Jonathan M. Blaxill, John P. Greenwood, Abdul M. Mozid, Jennifer A. Rossington, Murugapathy Veerasamy, Stephen B. Wheatcroft, Nick Curzen, Heerajnarain Bulluck

**Affiliations:** 1 Yorkshire Heart Centre, Leeds General Infirmary, Leeds Teaching Hospitals NHS Trust, Leeds LS1 3EX, UK; 2 Leeds Institute of Cardiovascular and Metabolic Medicine, University of Leeds, Leeds LS1 3EX, UK; 3 Faculty of Medicine, University of Southampton, Southampton SO17 1BJ, UK; 4 Coronary Research Group, University Hospital Southampton NHS Trust, Southampton SO17 1BJ, UK

**Keywords:** fractional flow reserve, angiography, coronary artery disease, percutaneous coronary intervention, coronary artery bypass graft surgery

## Abstract

Background and Aims: Randomised controlled trials (RCTs) comparing outcomes after fractional flow reserve (FFR)-guided versus angiography-guided management for obstructive coronary artery disease (CAD) have produced conflicting results. We investigated the efficacy and safety of an FFR-guided versus angiography-guided management strategy among patients with obstructive CAD. Methods: A systematic electronic search of the major databases was performed from inception to September 2022. We included studies of patients presenting with angina or myocardial infarction (MI), managed with medications, percutaneous coronary intervention, or bypass graft surgery. A meta-analysis was performed by pooling the risk ratio (RR) using a random-effects model. The endpoints of interest were all-cause mortality, MI and unplanned revascularisation. Results: Eight RCTs, with outcome data from 5077 patients, were included. The weighted mean follow up was 22 months. When FFR-guided management was compared to angiography-guided management, there was no difference in all-cause mortality [3.5% vs. 3.7%, RR: 0.99 (95% confidence interval (CI) 0.62–1.60), *p* = 0.98, heterogeneity (I^2^) 43%], MI [5.3% vs. 5.9%, RR: 0.93 (95%CI 0.66–1.32), *p* = 0.69, I^2^ 42%], or unplanned revascularisation [7.4% vs. 7.9%, RR: 0.92 (95%CI 0.76–1.11), *p* = 0.37, I^2^ 0%]. However, the number patients undergoing planned revascularisation by either stent or surgery was significantly lower with an FFR-guided strategy [weighted mean difference: 14 (95% CI 3 to 25)%, *p* =< 0.001]. Conclusion: In patients with obstructive CAD, an FFR-guided management strategy did not impact on all-cause mortality, MI and unplanned revascularisation, when compared to an angiography-guided management strategy, but led to up to a quarter less patients needing revascularisation.

## 1. Introduction

Current guidelines recommend fractional flow reserve (FFR) to guide revascularisation for intermediate stenoses with no prior evidence of myocardial ischaemia on non-invasive testing and in the setting of multivessel coronary disease [1]. These recommendations are predominantly based on the FAME (Fractional Flow Reserve Versus Angiography for Multivessel Evaluation) trial, which demonstrated lower rates of major adverse cardiac events (MACE), predominantly driven by repeat revascularisation, in patients with multivessel disease who had FFR-guided revascularisation, compared to angiography-guidance [2].

Subsequent randomised controlled trials (RCTs), performed in a variety of clinical settings, comparing outcomes after FFR-guided versus angiography-guided revascularisation have produced conflicting results, but have, in general, failed to demonstrate the expected additional benefit from using FFR in addition to angiography to guide diagnosis, management and revascularization [2,3,4,5,6,7,8]. Most recently, the FRAME-AMI trial (FFR- vs. Angiography-guided PCI in AMI with multivessel disease) found lower MACE with FFR-guided complete revascularisation, compared to angiography-guided, among 562 patients with ST-segment elevation myocardial infarction (STEMI) who had been committed to complete revascularisation of non-culprit coronary disease [3]. Furthermore, RIPCORD-2 (Does Routine Pressure Wire Assessment Influence Management Strategy at Coronary Angiography for Diagnosis of Chest Pain?) randomised 1100 patients undergoing diagnostic angiography for stable angina or non-ST elevation MI (NSTEMI) to either angiographic diagnosis and management alone, of angiography plus FFR assessment of all major coronary arteries. It found no difference in MACE, cost, or quality of life between the groups [4].

Given the discrepant outcome data, the aim of this study was to perform a contemporary meta-analysis of RCTs (including patients with stable angina or AMI, managed with medications, percutaneous coronary intervention (PCI) or coronary artery bypass grafts surgery (CABG), to compare clinical outcomes after an FFR-guided versus an angiography-guided management strategy in patients with obstructive CAD. 

## 2. Methods

This study was performed following the PRISMA (Preferred Reporting Items for systematic Reviews and Meta-Analyses) guidelines [9]. The protocol for this study was registered with the International Prospective Register of Systematic Reviews (PROSPERO ID: CRD42022356766).

### 2.1. Search Strategy

A systematic search of the online databases Medline and Embase via Ovid was performed, from inception to September 2022. Peer-reviewed RCTs were selected using combinations of the following keywords: ‘fractional flow reserve’; ‘pressure wire’; ‘FFR’; ‘coronary angiogram’; and ‘coronary angiography’. The electronic database search was supplemented by using the clinical trial registry ‘ClinicalTrials.gov’, to identify other relevant studies. The reference lists of included trials were also reviewed, for other appropriate trials. For completeness, we searched conference abstracts from recent major cardiology meetings, specifically the European Society of Cardiology, EuroPCR, Transcatheter Cardiovascular Therapeutics, the American College of Cardiology and the American Heart Association. Two investigators (C.J.M and A.M) independently screened abstracts against eligibility criteria. In case of discrepancies among the two independent investigators, a third independent investigator (H.B) was available to review the data, to resolve discrepancies by consensus among the investigators.

### 2.2. Eligibility Criteria

We included all RCTs comparing an FFR-guided versus an angiography-guided management strategy in patients with obstructive CAD, and reporting outcomes on death, MI and unplanned revascularisation. For studies with multiple publications, we used data from the longest reported follow-up. We included RCTs in which patients presented with either stable coronary artery disease, or acute coronary syndrome (ACS), including RCTs assessing non-infarct related artery stenoses following revascularisation of the culprit vessel in STEMI. RCTs of patients undergoing revascularisation with either coronary artery bypass graft surgery or percutaneous coronary intervention were included. Non-randomised trials, publications not in English, and those not reporting clinical outcomes of interest were excluded. We also excluded studies that used an FFR cut-off other than ≤0.8 to define significant ischaemia, because 0.8 is the FFR threshold accepted by international clinical guidelines for defining haemodynamically significant lesions [1].

### 2.3. Data Extraction

Baseline demographic and clinical outcome data were extracted from the main study reports. Supplementary material was also reviewed. Clinical outcome data were extracted on an intension-to-treat basis. For RCTs including an all-comer population undergoing angiography, we only included outcomes on the subsets with obstructive CAD.

### 2.4. Quality Assessment

**Figure 1:**
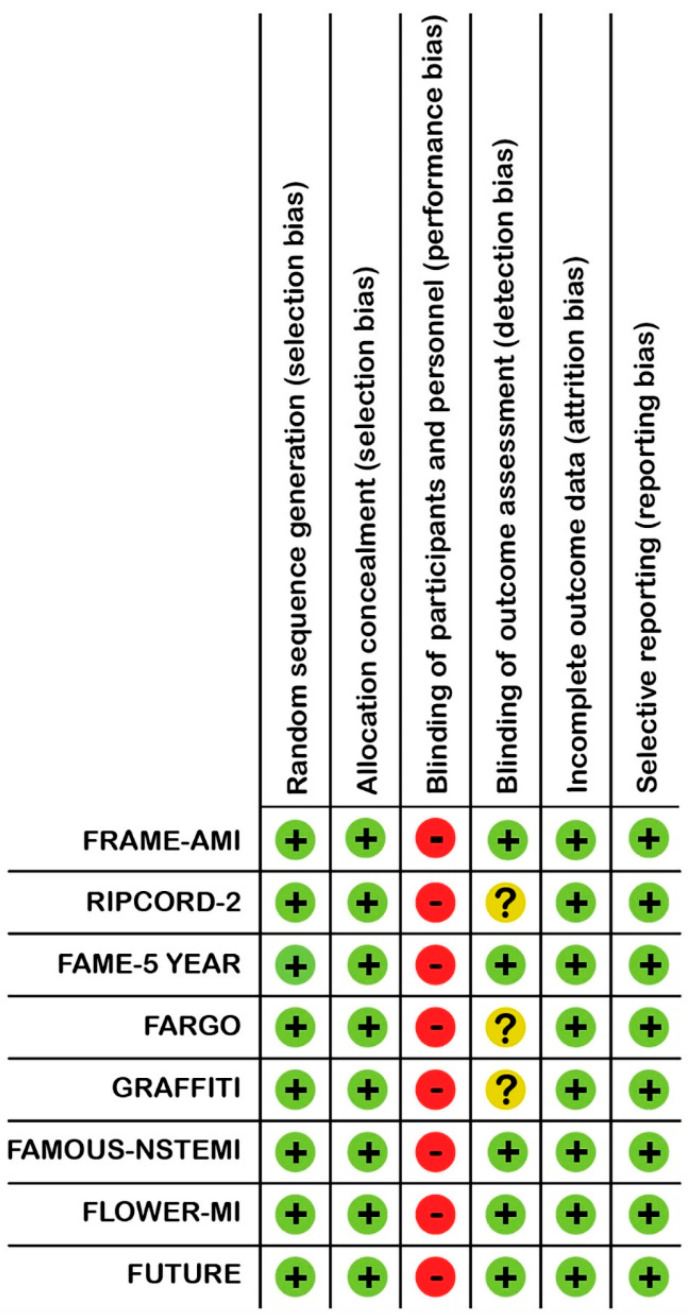
Risk of bias summary for the individual studies, by Cochrane risk assessment tool. + = low risk of bias, − = risk of bias, ? = unclear.

We assessed the risk of bias and the quality of included studies, according to the Cochrane risk of bias assessment tool [10] (Figure 1).

### 2.5. Outcomes

The main endpoints of interest were all-cause mortality, MI and unplanned revascularisation. We also investigated the number of stents implanted and number of patients proceeding to revascularisation in each group. We originally planned to stratify the results according to patients presenting with stable CAD or ACS, but outcome data for these individual endpoints were not available from the trial-level data. However, we were able to provide the pooled, trial-defined major composite endpoint analysis, stratified by stable CAD or ACS if available, from the selected RCTs.

### 2.6. Statistical Analysis

Weighted mean follow-up duration was calculated according to study size. We summarised the estimate of effect incorporating the clinical outcome as the risk ratio (RR) with 95% confidence intervals (CI). The pooled RR was calculated with a random-effects model, due to anticipated heterogeneity between included RCTs, using the Mantel-Haenszel method. We performed heterogeneity testing with Higgins I^2^, with a threshold of >50% suggestive of significant heterogeneity [11]. The statistical analyses were performed using Review Manager (RevMan) [Computer program]. Version 5.4, The Cochrane Collaboration, 2020.

## 3. Results

**Figure 2:**
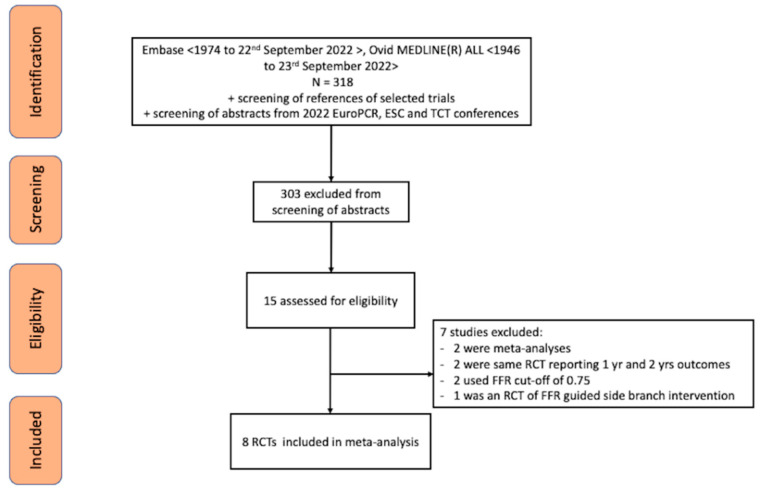
PRISMA diagram of the trial selection process.

Figure 2 shows the process of trial selection. Eight RCTs met the eligibility criteria [2−8,12], including a total of 5077 patients, with 1 of those [3] only recently presented in detail at the recent ESC 2022 conference and not yet published in full text. Among these, 2544 patients were in the FFR-guided group and 2533 were in angiography-guided group. Out of the included RCTs, five had follow-up of 1 year [4,6−8,12], one had follow up of 6 months [5], and two RCTs had longer follow-up of 3.5 [3] and 5 years [2]. The weighted mean follow-up was 22 months. Overall, the loss to follow-up of patients in this study was <1%.

### 3.1. Characteristics of Included RCTs

There was heterogeneity of clinical presentations included in the trials, endpoint definitions and treatment with CABG or PCI (Table 1 and Table 2). The trial-defined composite endpoint was not uniform in the RCTs, as highlighted in bold in Table 1. Revascularisation was exclusively with CABG in two RCTs (FARGO [Fractional Flow Reserve Versus Angiography Randomization for Graft Optimization] and GRAFFITI [Graft Patency After FFR-Guided Versus Angio-Guided CABG]) [5,6]. By contrast, revascularisation was exclusively with PCI in three RCTs (FRAME-AMI, FAME, FLOWER-MI [Flow Evaluation to Guide Revascularisation in Multivessel ST-elevation Myocardial Infarction]) [2,3,7]. Revascularisation was predominantly with PCI in the remaining three included trials (RIPCORD-2, FAMOUS-NSTEMI [Fractional Flow Reserve Versus Angiographically Guided Management to Optimise Outcomes in Unstable Coronary Syndromes], FUTURE [Functional Testing Underlying Coronary Revascularisation]) [4,8,12]. In general, there was low risk of bias across the included RCTs (Figure 1).

### 3.2. Baseline Characteristics of the Population

Trial characteristics are displayed in Table 1. Population and procedural characteristics are displayed in Table 2. The mean age of the entire population was 64 years and 81% were men. Overall, 39% of patients presented with stable CAD, whereas 61% presented with ACS. Twenty five percent of the population had diabetes mellitus. 

### 3.3. Clinical Endpoints

**Figure 3:**
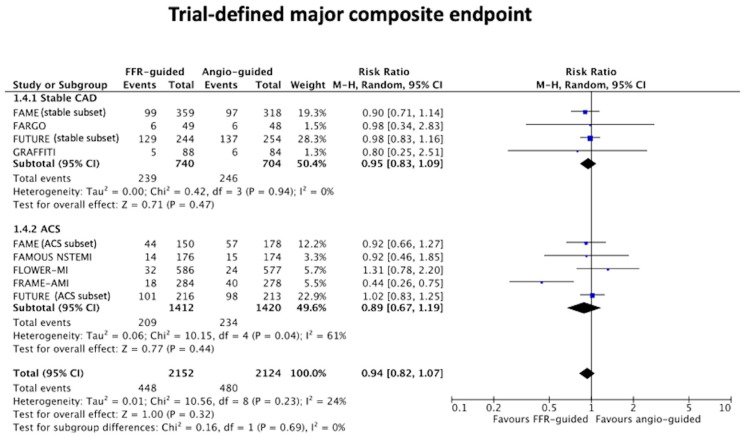
Forest plot of the trial-defined composite endpoint stratified by stable CAD and ACS.

There was no difference in the trial-defined composite endpoint, when stratified according to either stable CAD [32% vs. 35%, RR: 0.95 (95%CI 0.83 to 1.09), *p* = 0.47, I^2^, 0% or ACS 15% vs. 16%, RR: 0.89 (95%CI 0.67 to 1.19), *p* = 0.44, I^2^ 61%, between an FFR-guided group and the angiography-guided group (Figure 3).

**Figure 4:**
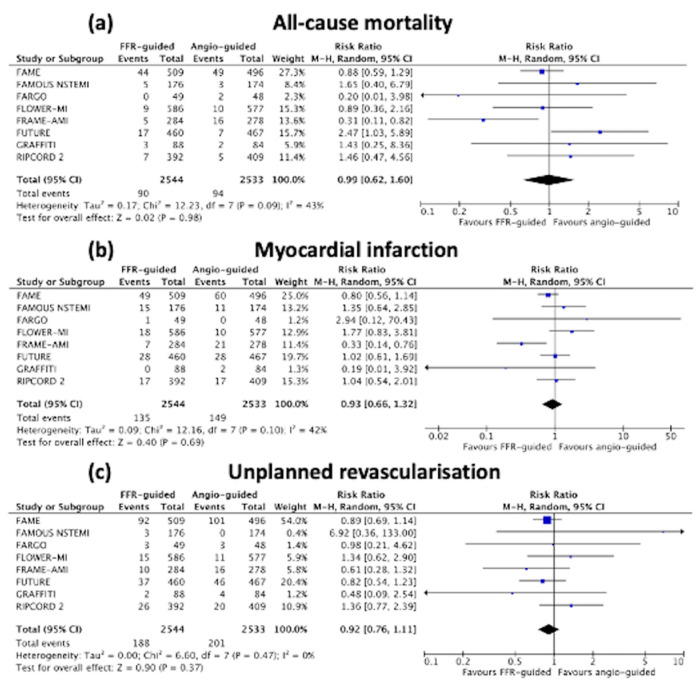
Forest plots of (**a**) all-cause mortality; (**b**) non-fatal myocardial infarction; and (**c**) unplanned revascularisation.

There was no difference in all-cause mortality between the FFR-guided group and the angiography-guided group [3.5% vs. 3.7%, RR: 0.99 (95% confidence interval (CI) 0.62 to 1.60), *p* = 0.98, I^2^ 43%, Figure 4a.

There was also no difference in non-fatal MI [5.3% vs. 5.9%, RR: 0.93 (95%CI 0.66 to 1.32), *p* = 0.69, I^2^ 42%, Figure 4b] or unplanned revascularisation [7.4% vs. 7.9%, RR: 0.92 (95%CI 0.76 to 1.11), *p* = 0.37, I^2^ 0%, Figure 4c] between the FFR- versus angiography-guided groups. 

Sensitivity analyses conducted via a leave-one-out meta-analysis did not change the statistical significance of the results.

### 3.4. Revascularisation and Stent Implanted per Allocated Strategy

**Figure 5:**
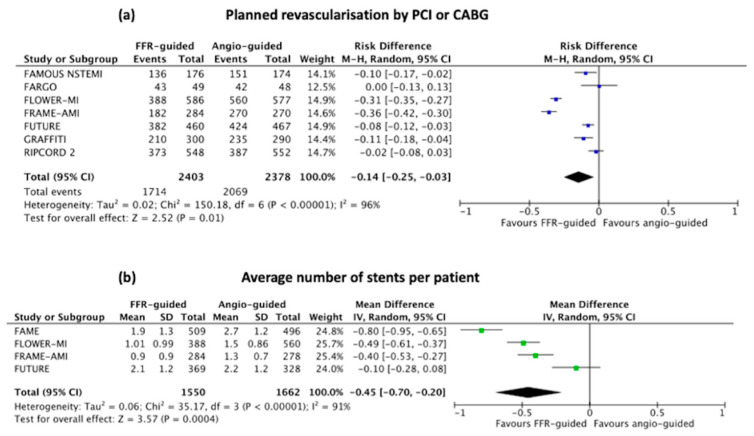
Forest plots of (**a**) average number of stented implanted; and (**b**) percentage of patients undergoing planned revascularisation as per their randomisation group.

The number patients undergoing planned revascularisation by either stent or surgery was significantly lower in the FFR-guided group [weighted mean difference: 14 (95% CI: 3 to 25)%, *p* ≤ 0.001], Figure 5a, when compared to the angiography-guided revascularisation strategy.

The pooled average number of stents was significantly lower in the FFR-guided group compared to the angiography-guided group [mean difference −0.45 (95%CI −0.70 to −0.20), *p* = 0.004], Figure 5b.

## 4. Discussion

In this contemporary meta-analysis of RCTs comparing FFR-guided (using a cut-off of ≤0.80) to angiography-guided management strategy for obstructive CAD, we found no difference in mortality, MI, or unplanned revascularisation, between the 2 strategies. However, an FFR-guided approach was associated with a lower number of patients who underwent revascularisation by up to a quarter (upper limit of the 95% CI). The latter finding is of considerable importance, highlighting the benefit to patients and the local health resource of such an approach. In fact, our findings could be summarised as follows: despite reducing the number of patients requiring revascularisation by up to 25%, an FFR-guided management strategy has no penalty in terms of the rate of adverse clinical events.

Two previous meta-analyses included 5094 patients from 7 RCTs [13], and an analysis from 5 RCTs totalling 2288 patients [14]. Both of these meta-analyses [13,14] found no difference in mortality when FFR-guidance was compared to angiography-guidance for complete revascularisation. However, our study also includes FRAME-AMI [3], only recently reported. We also excluded the RCT by Quintella et al. [15] (n = 69), which was included in the previous meta-analysis [14], and the DEFER-DES trial [16] (Fractional Flow Reserve to Determine the Appropriateness of Angioplasty in Moderate Coronary Stenosis), which was included in the larger prior meta-analysis [13], as they used an FFR threshold of <0.75. Furthermore, we excluded the DK-CRUSH VI trial (Double Kissing Crush Versus Provisional Stenting Technique for Treatment of Coronary Bifurcation Lesions) [17], which was included in the larger prior meta-analysis [13], as only the side branch involved in a provisional bifurcation stenting strategy was randomised to either FFR-guided or angiography-guided revascularisation, rather than the lesion in the main vessel. Furthermore, we took care to only include patients with obstructive CAD from the RIPCORD-2 trial [4], to better reflect current clinical practice of when FFR use would be considered, which was not the case in the previous meta-analysis [13]. Lastly, we also included trials with CABG as the revascularisation strategy, which makes our findings more relevant to everyday clinical practice, and our meta-analysis builds on the previous work by Matthews et al. [18], which only included 3 trials [7,8,19] of patients undergoing PCI only and clinical outcomes were limited to 1 year.

The role of FFR in acute MI setting has been a subject of debate. FFR is usually performed in the non-infarct related artery in the setting of STEMI rather that the infarct related artery. Therefore, the impact of acute infarct and edema is minimal. However, STEMI patients may have caffeine or caffeine-containing product on board, and therefore FFR done during the index procedure would have a high false-negative rate. In NSTEMI, FFR can be done both in the infarct-related artery and the non-infarct related artery as shown in FAMOUS-NSTEMI but was not powered for clinical outcomes. One would expect that if a NSTEMI patient with a large infarct size or area of edema, FFR could be falsely negative in view of the inability of that infarct related territory to reach maximum hyperemia. We did attempt to stratify the trial-defined MACE by clinical presentation and in view of the inherent limitation of doing FFR in acute MI setting, although there was no difference in MACE between the 2 groups, the heterogeneity was high at 61%. Further studies are required to confirm the role of FFR in acute MI setting. 

It is well known that discrepancy exists between angiographic visual estimates of stenosis severity, and physiologically significant flow limitation that causes downstream myocardial ischaemia [20]. The prevalence of a discordance between the visual estimate of stenosis significance and FFR measurement is between 20–30% of all lesions, and this mismatch involves lesions as little as 30% stenosis by eye and above 90% [4,21]. The absence of myocardial ischaemia is associated with excellent outcomes using optimal medical therapy [22] and FFR is regarded as the reference standard invasive method to define lesion-specific ischaemia [23]. It has been logically been suggested that judgements based on angiographic visual estimates of lesion severity are subjective, potentially leading to misdiagnosis and unnecessary stent implantation or even CABG, with the possibility of procedure-related complications, leading to worse outcomes [20]. In contrast, our findings demonstrate that guiding revascularisation in a range of clinical scenarios encountered in our daily practice based on angiography alone, without FFR, does not adversely impact on major adverse ischaemic events. One potential explanation for our findings might be the impact of vulnerable plaque characteristics, which have been shown to be associated with adverse outcomes [24] and could potentially exist in lesions without significant ischaemia. Most recently, this was demonstrated in the COMBINE OCT-FFR trial (Combined Optical Coherence Tomography Morphologic and Fractional Flow reserve Haemodynamic Assessment of Non-Culprit Lesions to Better Predict Adverse Event outcomes in Diabetes Mellitus patients) [25]. COMBINE OCT-FFR found that, among diabetic patients with ≥1 FFR-negative lesions, thin-cap fibroatheroma detected on optical coherence tomography was associated with a five-fold higher rate of MACE, despite the absence of ischaemia [25]. Therefore, there has recently been a paradigm shift in our understanding that plaque burden (the higher the plaque burden, the more likely for vulnerable plaques to develop), may impact on hard clinical outcomes irrespective of the physiological significance of lesions. Further studies are warranted to improve understanding of whether revascularization decisions could be improved by assessment using the combination of both plaque vulnerability with OCT and physiological lesion significant with FFR The INTERCLIMA trial (Interventional Strategy for Non-culprit Lesions with Major Vulnerability Criteria at OCT in Patients with ACS) (NCT05027984), PREVENT trial (The Preventive Coronary Intervention on Stenosis With Functionally Insignificant Vulnerable Plaque) (NCT02316886) and COMPARE STEMI ONE trial (Comparison Of Reduced DAPT Followed by P2Y12 Inhibitor Monotherapy With Prasugrel vs. standard Regimen in STEMI Patients Treated With OCT-guided vs. aNgio-guided complete Revascularisation) (NCT05491200), are currently ongoing, to assess whether an imaging-guided approach to identify vulnerable plaques, would improve clinical outcomes.

There are a number of limitations to our study. Firstly, it is an aggregate of trial-level data, rather than individual patient-level data. Therefore, we could not perform in-depth sub-group analyses, stratified by diabetes status, clinical presentation, or treatment with CABG. We cannot exclude the possibility that heterogeneity of the populations, for example the prevalence of diabetes, may have influenced the conclusions. Nonetheless, evidence suggests that robustly performed trial-level meta-analyses often produce similar conclusions to patient-level meta-analyses [26]. We did provide subgroup analysis for the trial-defined composite endpoint for patients presenting with stable CAD or ACS and our findings were similar to those observed for all the RCTs were pooled together. Secondly, there was heterogeneity across the included RCTs, with respect to inclusion criteria, primary endpoints, and follow up duration. Of note, RIPCORD-2 [4], GRAFFITI [6] and FAMOUS-NSTEMI [12] included lesions with 30% angiographic stenosis assessed visually, compared to 50% in the other studies included in our meta-analysis [2,3,5,7,8]. The percentage of patients with ACS was lowest in the GRAFFITI trial [6]. In contrast, FLOWER-MI [7] exclusively included STEMI patients with bystander disease, and FRAME-AMI [3] only included patients with STEMI or NSTEMI. FAMOUS-NSTEMI [12] exclusively included patients with NSTEMI. Approximately half of the patients included in RIPCORD-2 [4] and FUTURE [8] presented with ACS. However, these studies reflect the patient population we would encounter in our clinical practice for pressure wire use to guide treatment. It should also be noted that the indication for FFR use to guide PCI is more widely clinically applicable whereas the aim of FFR use in the 2 RCTs to guide CABG (FARGO and GRAFFITI trials) was to assess graft patency post-surgery. Therefore, FFR use in those already planned for CABG is less clinically applicable at present, pending further adequately powered RCTs for hard clinical outcomes in CABG patients and is a limitation of our study.

In conclusion, this contemporary meta-analysis shows that an FFR-guided management strategy did not impact on all-cause mortality, MI and unplanned revascularisation, when compared to an angiography-guided management strategy, after a weighted mean follow-up of 22 months. However, an FFR-guided approach led to up to 1 in 4 less patients needing revascularisation, which has important benefits to patients and the local provision of health resources. 

## Figures and Tables

**Table 1:** Characteristics of RCTs, comparing FFR with angiography, for guiding revascularisation.

Study	Year Published	Enrolment Centres	Participants and Presentation	Primary Endpoint	Follow-Up (Years)	Loss to Follow- Up, n (%)	Findings
FRAME-AMI [3] (NCT02715518)	2022	14 sites in Korea	562 patients (STEMI/ NSTEMI)	MACE defined as the composite of death, MI, or unplanned revascularisation	3.5	0.4	Lower composite rates of death, MI, or unplanned revascularisation with FFR-guidance vs. angiography-guidance (7.4% vs. 19.7%, hazard ratio: 0.43 [95% CI: 0.25, 0.75] *p* = 0.003)
RIPCORD-2 [4] (NCT02892903)	2022	17 sites in United Kingdom	1100 patients (stable angina/ NSTEMI)	Total hospital cost and quality of life	1	0.3	No difference in median hospital costs or quality of life for FFR-guidance vs. angiography-guidance. No difference in the composite of death, stroke, MI, or unplanned revascularisation for FFR-guidance vs. angiography-guidance (9.5% vs. 8.7%, *p* = 0.064).
FAME 5 year [2] (NCT00267774)	2015	20 sites in the United States and Europe	1005 patients (stable/unstable Angina)	MACE defined as the composite of death, MI, or unplanned revascularisation	5	7.5	At 5 years, no difference in the composite of death, MI, or unplanned revascularisation with FFR -guidance vs. angiography-guidance (28% vs. 31%, relative risk: 0.91 [95% CI: 0.75, 1.10] *p* = 0.31). At 2 years, MACE was lower with FFR-guidance vs. angiography-guidance. Number of stents implanted per patient was lower with FFR-guidance vs. angiography-guidance (mean 1.9 ± 1.3 vs. 2.7 ± 1.2, *p*< 0.0001).
FARGO [5] (NCT02477371)	2018	3 sites in Denmark	100 patients (stable angina/ NSTEMI)	Graft failure in the percentage of all grafts	0.5	0.0 (for MACE) 25.0 (for angiogram follow-up at 6 months)	No difference in graft failure rates with FFR guidance vs. angiography-guidance (16% vs. 12%, *p* = 0.97). No difference in the composite of death, nonprocedural MI, unplanned revascularisation and stroke with FFR-guidance vs. angiography-guidance (12% vs. 12%, *p* = 0.97).
GRAFFITI [6] (NCT01810224)	2019	6 sites in Europe	172 patients (stable angina/ NSTEMI)	Graft occlusion	1	1.7 (for MACE) 35.5 (for Coronary imaging follow- up at 6 months)	No difference in graft failure rates with FFR- guidance vs. angiography-guidance (19% vs. 20%, *p* = 0.885). No difference in the composite of death, MI, unplanned revascularisation and stroke with FFR-guidance vs. angiography-guidance (3.7% vs. 7.1%, hazard ratio: 1.28 [95% CI: 0.39, 4.16], *p* = 0.687).
FAMOUS- NSTEMI [12] (NCT01764334)	2014	6 sites in the United Kingdom	350 patients (NSTEMI)	Proportion of patients allocated to medical management	1	0.0	Higher proportion of patients initially treated by medical therapy with FFR-guidance vs. angiography guidance (22.7% vs. 13.2%, *p* = 0.022). No difference in the composite of cardiovascular death, MI, or unplanned hospitalisation for heart failure (8.0% vs. 8.6%, risk difference −0.7% [95% CI: −6.7, 5.3%] *p* = 0.89).
FLOWER-MI [7] (NCT02943954)	2021	41 sites in France	1163 patients (STEMI)	MACE defined as the composite of death, MI, and unplanned hospitalisation leading to urgent revascularisation	1	0.4	At 5 years, no difference in the composite of death, MI and urgent revascularisation with FFR- guidance vs. angiography-guidance (5.5% vs. 4.2%, hazard ratio: 1.32 [95% CI: 0.78, 2.23] *p* = 0.31).
FUTURE [8] (NCT01881555)	2021	31 sites in France	927 patients (stable angina/ ACS/atypical chest pain/ silent ischaemia)	Composite of death, MI, stroke or unplanned revascularisation	1	0.1	No difference in the composite of death, MI, stroke or unplanned revascularisation with FFR-guidance vs. angiography-guidance (14.6% vs. 14.4%, hazard ratio: 0.97 [95% CI: 0.69, 1.36], *p* = 0.85).

Abbreviations: CI = confidence interval; FAME = Fractional Flow Reserve Versus Angiography for Multivessel Evaluation; FAMOUS-NSTEMI = Fractional Flow Reserve Versus Angiographically Guided Management to Optimise Outcomes in Unstable Coronary Syndromes; FARGO = Fractional Flow Reserve Versus Angiography Randomisation for Graft Optimisation; FFR = fractional flow reserve; FLOWER-MI = FLOW Evaluation to Guide Revascularisation in multivessel ST-elevation Myocardial Infarction; FRAME-AMI = FFR vs. Angiography-guided PCI in AMI with multivessel disease; FUTURE = Functional Testing Underlying Coronary Revascularisation; GRAFFITI = Graft Patency After FFR-Guided Versus Angio-Guided CABG; MACE = major adverse cardiac events; NSTEMI = non-ST segment elevation myocardial infarction; RIPCORD-2 = Does Routine Pressure Wire Assessment Influence Management Strategy at Coronary Angiography for Diagnosis of Chest Pain?; STEMI = ST segment elevation myocardial infarction.

**Table 2:** Patient and procedural characteristics from RCTs, comparing FFR with angiography only, for guiding revascularisation.

Study	Strategy	Age, Years (Mean ± SD, or Median [IQR])	Male (%)	Diabetes Mellitus (%)	Smoker (%)	ACS Presentation (%)	Treatment with CABG (%)	Procedure Time for PCI, Mins (Mean ± SD, or Median IQR])	FFR Cut-Off	Angiogram Visual Stenosis Threshold for PCI (%)
FRAME-AMI [3] (NCT02715518)	Angio (n = 278)	62.7 ± 11.5	84.2	30.9	37.8	100.0	0	Not reported	NA	>50
	FFR (n = 284)	63.9 ± 11.4	84.5	34.2	32.0	100.0	0	Not reported	≤0.8	NA
RIPCORD-2 [4] (NCT02892903)	Angio (n = 552)	64.3 ± 10.2	77.2	17.6	65.0	53.1	9.2	42.4 ± 27.0	NA	≥30
	FFR (n = 548)	64.3 ± 10.0	73.5	20.6	58.5	50.4	11.9	69.0 ± 27.0	≤0.8	NA
FAME 5 year [2] (NCT00267774)	Angio (n = 496)	63.9 ± 10.0	74.0	25.0	30.0	31.3	0	70.0 ± 44	NA	>50
	FFR (n = 509)	64.5 ± 10.4	75.0	22.0	25.0	25.1	0	71.0 ± 43	≤0.8	NA
FARGO [5] (NCT02477371)	Angio (n = 48)	65.3 ± 8.8	89.0	23.0	17.0	14.0	100.0	NA	NA	≥50
	FFR (n = 49)	66.4 ± 6.4	88.0	22.0	27.0	31.0	100.0	NA	≤0.8	NA
GRAFFITI [6] (NCT01810224)	Angio (n = 84)	67 (63, 72)	79.00	40.0	42.0	11.0 (for entire population)	100.0	NA	NA	≥30
	FFR (n = 88)	67 (62, 72)	83.0	35.0	53.0		100.0	NA	≤0.8	NA
FAMOUS- NSTEMI [2] (NCT01764334)	Angio (n = 174)	61.6 ± 11.1	73.0	14.9	40.8	100	6.9	70.5 ± 33.5	NA	≥30
	FFR (n = 176)	62.3 ± 11.0	75.6	14.8	40.9	100	6.2	66.5 ± 23.4	≤0.8	NA
FLOWER-MI [7] (NCT02943954)	Angio (n = 577)	61.9 ± 11.4	81.1	14.2	36.4	100	0	32.0 (20.0, 24.0)	NA	≥50
	FFR (n = 586)	62.5 ± 11.0	85.0	18.3	40.1	100	0	31.0 (21, 45)	≤0.8	NA
FUTURE [8] (NCT01881555)	Angio (n = 467)	66.0 ± 11.0	82.0	32.0	26.0	46.0	12.0	Not reported	NA	≥50
	FFR (n = 460)	65.0 ± 10.0	85.0	31.0	24.0	47.0	12.0	Not reported	≤0.8	NA

Abbreviations: ACS = acute coronary syndrome; CABG = coronary artery bypass graft surgery; FFR = fractional flow reserve; IVUS = intravascular ultrasound; IQR = interquartile range; NA = not applicable; OCT = optical coherence tomography; PCI = percutaneous coronary intervention; SD = standard dev.

## Data Availability

The data underlying this article are available in the article.
